# "Ierne" and Nurses All

**Published:** 1918-07-06

**Authors:** 


### A NEW CHARTER OF LIBERTY.

### "Ierne" and Nurses All.

The thanks of the profession are due to Lord Knutsford for his interesting and useful contribution to the Liberty of the Nurse question. His lordship tilts his lance with skill, and having regard to the fact that he bears on his shoulders the responsibility of the "London" it would perhaps be scarcely fair to blame him for persistently begging the question why a hospital nurse, above other workers, should be called upon to forfeit her freedom. For, of course, to say that "she agrees" is obviously begging the question. What I hoped Lord Knutsford would explain was why she should be asked to sign such an agreement.

However, although Lord Knutsford avoids the direct issue, and boldly shakes hands with Shylock, nevertheless he makes plain at least a part of the operating cause. Public hospitals, even the best, it may be read between the lines, are inadequately equipped with funds, or with accommodation, or both. This is a part of the reason for harsh conditions and little time to play. The other reason is an unorganised and inarticulate profession whose members have to take what they are offered. Let the unsophisticated nurse bear in mind that no Registration Act will ever prolong her holidays by an hour, or add one stiver to her pay. This by the way. What I desire to emphasise is that the hospital owes the nurse more than she receives in payment. She is sacrificed in the cause of the nation's charity, and any recompense that is made to her in the future can never be described as eleemosynary, but rather as the payment of a debt. It is extremely important that this fact should be generally recognised, and more especially by chairmen and boards of governors of hospitals, who should lend their aid in establishing British nurses in a position worthy of a rich and powerful nation on the one hand, and of faithful and devoted public servants on the other. Public hospital duty is State duty, and if the nurse is not fairly and even generously dealt with by voluntary effort, the only alternative is an appeal for State aid, which, of course, involves State interference with its accompanying disadvantages. "It seems to me utterly inexcusable," says Lord Knutsford, "to run a hospital at the expense of its nurses." More or less they are all so run, including even the "London." But hospital boards and chairmen are not, therefore, necessarily tyrants or slave drivers. As Lord Knutsford, in effect, expresses it—"the difficulties are great, and I have to find the money." It is a human attitude, but the nurse is the victim. Now I desire to return to the genesis of this discussion, and to ensure, if possible, that it shall not be consigned to the rubbish-heap of things unaccomplished. Lord Knutsford was one of the first men in England to conceive the idea of a Charter of Liberty for Nurses. He modestly protests that probably the title was a silly one. Not so. In the title is wrapped up everything: the remainder of it we shall all forget. On a Charter of Liberty is built up the whole fabric of British freedom, in defence of which we are spending untold wealth of manhood and of gold. That Charter was drawn up, not by the recreant king who signed it, but by his subjects, and its clauses embodied what British men and women regarded as the essential minimum to make life worth living. What sayeth the reader to a Charter of Liberty for Nurses drawn up by themselves? If such an adventure accomplished naught else, it would at least be an experiment in articulation. The song to which the poet hearkened in the woodland consisted of "a thousand blended notes." Can we not have such a blend? We have heard much, perhaps a little too much, about a "one-portal examination," and much also of "registration" and "legislation"—not one tiny atom of which, in my humble opinion, the working nurse ever articulated. I venture to assert that on the subject of off-duty privileges, including an annual holiday, every nurse in the kingdom holds views specific and pronounced, but so far unexpressed. I suggest that a collective opinion voluntarily offered by, say, a thousand nurses, would materially help to advance the cause of reform.

I shall accordingly be glad if those who agree will send a postcard addressed "Ierne," Offices of The Hospital, 28-29 Southampton Street, Strand, London, W.C. 2, headed on the back—"A Nurse's Charter of Liberty," and setting out thereunder what each regards as a reasonable and proper allowance of off-time, adding at the foot (not for publication) the name and address of the writer and her rating in nursing. If one writer expresses an opinion on behalf of many, let the number so represented be stated. The writer should be a hospital nurse of any grade from probationer up. I propose on receipt of these postcards to analyse their contents and to announce the composite result, and so be able to compare the standpoint and view of the worker with that of the employer. If the number of entries is large, the result ought to be of immediate and historic interest.

IERNE.

